# Gas Vesicle Nanoparticles for Antigen Display

**DOI:** 10.3390/vaccines3030686

**Published:** 2015-09-07

**Authors:** Shiladitya DasSarma, Priya DasSarma

**Affiliations:** Department of Microbiology and Immunology, Institute of Marine and Environmental Technology, University of Maryland, Baltimore, MD 21202, USA; E-Mail: pdassarma@som.umaryland.edu

**Keywords:** protein nanoparticle, adjuvant, carrier, SIV, *Chlamydia*, *Salmonella*, typhoid, *Plasmodium*, malaria, luciferase

## Abstract

Microorganisms like the halophilic archaeon *Halobacterium* sp. NRC-1 produce gas-filled buoyant organelles, which are easily purified as protein nanoparticles (called gas vesicles or GVNPs). GVNPs are non-toxic, exceptionally stable, bioengineerable, and self-adjuvanting. A large gene cluster encoding more than a dozen proteins has been implicated in their biogenesis. One protein, GvpC, found on the exterior surface of the nanoparticles, can accommodate insertions near the C-terminal region and results in GVNPs displaying the inserted sequences on the surface of the nanoparticles. Here, we review the current state of knowledge on GVNP structure and biogenesis as well as available studies on immunogenicity of pathogenic viral, bacterial, and eukaryotic proteins and peptides displayed on the nanoparticles. Recent improvements in genetic tools for bioengineering of GVNPs are discussed, along with future opportunities and challenges for development of vaccines and other applications.

## 1. Introduction

Nanoparticle-based vaccines, such as those employing gas vesicle nanoparticles (GVNPs), have certain potential advantages [1,2,3,4,5]. They offer reduced risk of infection compared to live-attenuated vaccines, and enhanced protection compared to subunit vaccines. Nanoparticles may simultaneously function as adjuvant and delivery system, by presenting antigens to the antigen presentation cells (APCs), and as immunopotentiators, increasing immunogenicity without adverse reactogenicity. Known properties of GVNP preparations from a salt-loving microbe, *Halobacterium* sp. NRC-1, a member of the third Domain, the Archaea, make these nanoparticles useful for antigen delivery and vaccine development [5].

The effectiveness of *Halobacterium* GVNPs as an adjuvant and antigen delivery system is underscored by both their stability and non-toxicity [5,6,7,8,9,10,11,12,13,14,15,16]. These nanoparticles are among the most resilient biological structures known, stabile for extended periods of time, even in the absence of a cold chain, and releasing displayed antigens slowly to the immune system. Moreover, GVNPs have no known toxic effects in animals, either systemically or at the site of administration. *Halobacterium* cells lack a bacterial-type cell envelope and are free of lipopolysaccharide (LPS), resulting in GVNP preparations free of the endotoxin. GVNPs are therefore biocompatible, and when administered by needle injection, subcutaneously (SC) or intraperitoneally (IP), elicit systemic immunity [5,6].

Over the past fifteen years, diverse antigens have been displayed on GVNPs, including short peptides and relatively large proteins, via fusion to a protein bound to the external surface of the nanoparticles, GvpC [5,6,7,8,9,10,11,12]. The source of antigens thus far have included a virus, the simian immunodeficiency virus (SIV) [6,7,8], two bacteria, the obligate intracellular pathogen *Chlamydia trachomatis* and facultative intracellular pathogen *Salmonella enterica* [9,10,11], and a eukaryote, the parasitic protozoan *Plasmodium falciparum* [12]. Antigenic proteins have ranged from secreted proteins, to coat and envelope proteins, and transcription factors, and the resulting nanoparticles have been studied both *in vitro* and *in vivo*. In one case, protection has been tested by challenge with the pathogen [11].

In this review, we cover genetic and biochemical studies of GVNPs primarily employing the *Halobacterium* sp. NRC-1 system. Although due to the extreme stability of the GVNP structure, characterization of these unique nanoparticles is still incomplete, the ability to bioengineer them for display of antigenic peptides and proteins has nevertheless advanced considerably. These efforts are reviewed here, along with improvements to tools for bioengineering of nanoparticles for vaccines, and a discussion of future opportunities and challenges.

## 2. Biology of GVNPs

GVNPs are commonly found in prokaryotic species inhabiting aquatic environments and have been reported widely in Archaea (haloarchaea and methanogens) and Bacteria (phototrophs and heterotrophs) [14,15,16]. In haloarchaea and cyanobacteria, gas vesicles are known to function in promoting cell buoyancy for vertical motility in aquatic environments, facilitating increased respiration, and phototrophic growth. However, these organelles are also found in anaerobic microorganisms, such as in methanogens [17,18] and soil bacteria [19,20] where their function is not clear.

**Figure 1:**
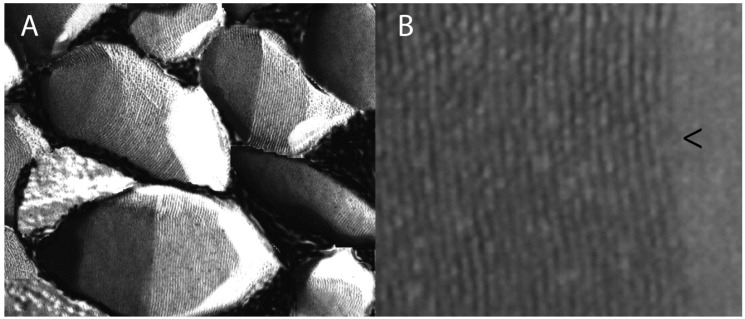
(**A**) Gas vesicle nanoparticles (GVNPs) produced in *Halobacterium* sp. NRC-1 observed by freeze-fracture electron microscopy; (**B**) Higher magnification shows the ribbed structure formed by a shallow spiral of the protein GvpA and stabilized by the bioengineerable protein GvpC. Predicted growth point in GVNP biogenesis is marked with an arrowhead.

GVNPs are easily purified by flotation after cell lysis due to their buoyancy. They have species-characteristic shape and morphology, ranging from 50 nm to over 1 μm in length, and from 30 to 250 nm in width (Figure 1) [14,21,22]. Narrower structures are more resistant to collapse when exposed to hydrostatic pressure and are generally observed in microorganisms at greater depths, while microorganisms in shallow pools contain predominantly wider forms, which, with fewer subunits, encompass a larger volume [23]. Strains capable of synthesizing both narrow cylindrical and wider spindle-shaped GVNPs may inhabit both shallow and deep brine pools [24,25,26].

GVNPs are composed of a thin lipid-free protein membrane 20 Å in thickness surrounding a gas-filled space [27,28,29,30]. The membrane is gas-permeable and allows the diffusion of many dissolved gases such as nitrogen, oxygen, carbon dioxide, and methane [31,32]. As a result, gases are reported to be in equilibrium with those found in the cytoplasm. GVNP biogenesis proceeds by growth from the tips, observed as small bi-cones, to progressively longer cylindrical structures [33]. The observed ribbed structures are formed from a shallow spiral of proteins [34]. During biogenesis, water is hypothesized to be excluded by hydrophobic forces at the inner surface of the membrane, with gases accumulating through passive diffusion [35].

Detailed analysis of GVNP biogenesis has suffered from extreme resistance of the constituent proteins to solubilization [29,36]. A single major protein, GvpA, was originally identified and hypothesized to serve as the sole protein constituting the membrane [14]. GvpA strongly self-associates and has been compared to amyloid proteins [37,38]. The second protein, GvpC, was discovered as a component of GVNPs from haloarchaea and cyanobacteria and, in *Halobacterium* sp. NRC-1, the GvpC gene has been bioengineered to display foreign sequences on the nanoparticles [5,6,7,8,9,10,11,12,39,40]. Additional studies have shown the involvement of more than a dozen genes in GVNP formation and at least seven proteins are present in the mature nanoparticles [13,15,41,42].

## 3. Gvp Gene Cluster

Complexity in the *gvp* genetic locus coding for gas vesicle proteins was found in *Halobacterium* sp. NRC-1 after the GvpA protein was cloned using the heterologous gene from *Calothrix* PCC 7601 as a probe [43,44,45]. In *Halobacterium* strains, the *gvp*A gene mapped to a megaplasmid, pNRC100, and subsequently pHH1 [43,46,47], and a second copy of the gene, named *gvp*A2 (also referred to as *gvp*B and c-vac), was identified later in a second megaplasmid, pNRC200 [26,46,48]. Analysis of gas vesicle-deficient mutants (Vac^−^) of *Halobacterium*, which occur spontaneously at a frequency of about 1%, revealed insertions in genes far upstream or downstream of *gvp*A [49,50]. Sequencing and transcript analysis surrounding the *gvp*A region of pNRC100 resulted in the finding of a total of 14 *gvp* genes, named *gvp*M, L, K, J, I, H, G, F, E, D, A, C, N, and O organized into two divergent operons [40,41]. A nearly identical arrangement of genes was observed in pHH1 [51] and pNRC200 [48].

Interestingly, while the *gvp* gene cluster was found to be highly conserved in *Halobacterium* strains, variations in gene content were reported in cyanobacteria and Gram-positive bacteria [15,19,20,52,53,54,55]. For example, *Anabaena flos-aquae*, a cyanobacterium, contained *gvp*A, C, N, J, K, and L/F, and *Bacillus megaterium*, a Gram-positive bacterium, contained *gvp*A, N, F, G, I, J, K, L, M, and O [19,56]. The genome sequence of *Streptomyces coelicolor* revealed a gene cluster with *gvp*A, O, F, G, J, K, L, and M [20] and the *Methanosarcina barkeri* genome contained *gvp*A, N, O, F, G, J, K, L, and M [57]. These findings all together have suggested that the *gvp*A, N, F/L, G, J, and K genes are found in nearly all gas vesicle gene clusters and may be essential for GVNP formation in diverse microorganisms. In addition, the *gvp*C gene was found to be present in both haloarchaea and cyanobacteria.

In *Halobacterium*, a naturally occurring *gvp*A mutant was characterized that did not produce GVNPs [43]. In order to determine which other *gvp* genes were required for GVNP production, the *gvp* gene cluster on pNRC100 was subjected to systematic mutagenic analysis [58,59]. A kanamycin resistance cassette (*kappa*) [42,58] was used to disrupt *gvp*M, L, K, J, I, H, G, F, E, D, C, N, and the N/O boundary, and all of the mutated derivatives, except for *gvp*M and the N/O boundary insertions, produced little or no wild-type GVNPs [42]. Polar effects were generally ruled out by deletion of an internal portion of the *kappa* cassette. Insertions in the *gvp*F, I, J, K, or L genes were found to produce no discernable wild-type GVNPs, while insertions into *gvp*G, or H, resulted in production of less than 1% of wild type GVNP levels, and insertions into *gvp*D or E resulted in low levels of GVNP production. Interestingly, insertions into *gvp*C and N produced GVNPs about one-third the length of the wild type, indicating that these genes acted in promoting growth of GVNPs but were not absolutely essential for their production.

Additional genetic studies on similar *gvp* gene clusters in *Halobacterium* megaplasmid pHH1 and in the *Haloferax mediterranei* chromosome resulted in findings that were largely consistent [52,60,61,62,63,64]. A deletion in the pHH1 *gvp*C gene resulted in irregularly shaped GVNPs, suggesting the involvement of GvpC in determining shape [62] in addition to assisting growth [42] and increasing stability [39]. Deletions of *gvp*D and E reduced but did not prevent GVNP production [62,63], confirming that they are not absolutely essential [42]. Additional studies indicated a role for *gvp*D and E in regulation of GVNP formation and essentiality of the terminal *gvp*M and *gvp*O genes [52,63,64,65,66,67,68]. Recently, overexpression of individual genes or pairs of genes in the gene cluster has suggested interactions between GvpM and GvpH, GvpJ, and GvpL [69,70]. In spite of these findings, many questions still remain regarding the roles of individual *gvp* genes in biogenesis of these buoyant nanoparticles, which are challenging due to the uniqueness and complexity of the *gvp* gene cluster.

## 4. Gas Vesicle Proteins

Due to the exceptional stability of the GVNP membrane, only two proteins, initially GvpA [43,71] and subsequently GvpC, were reported [39,40]. However, genetic analysis revealed the involvement of many other genes in the large *gvp* gene cluster of *Halobacterium* plasmid pNRC100 [72], and immunoblotting and mass spectrometric analysis showed 5–8 additional proteins to be present in nanoparticles of *Halobacterium* sp. NRC-1 (Table 1) [13,73]. GvpA was identified using a highly denaturing phenol-acetic acid-urea (PAU)-polyacrylamide gel and immunoblotting [40,74]. Two small acidic polypeptides similar to GvpA, GvpJ and GvpM (pfam741), were also identified using immunoblotting and confirmed by mass spectrometry [13,73]. The pfam741 family has been proposed to determine the curvature of the nanoparticle structure and/or to mediate the transition from cones to the cylindrical region. Spectroscopic and *de novo* modeling studies of GvpA have further suggested that this protein adopts a coil-α-β-β-α-coil fold with anti-parallel β-strands forming a hydrophobic surface on the internal face of the nanoparticles and a hydrophilic surface on the external side [75,76,77].

**Table 1:** Gas vesicle proteins and predicted proteins in *Halobacterium* sp. NRC-1.

Name	MW	Functions and Characteristics
GvpA	8005	Major gas vesicle protein with predicted coil-α-β-β-α-coil fold (pfam741)
GvpC	42,391	Minor gas vesicle protein with 8 repeats and acidic tail
GvpD	59,341	Probable regulatory protein with predicted NTP binding motif
GvpE	21,009	Probable regulatory protein with possible leucine zipper domain
GvpF	23,962	Minor gas vesicle protein with coiled-coil domain (pfam 6386)
GvpG	10,014	Minor gas vesicle protein
GvpH	19,883	Predicted gene product of unknown function
GvpI	16,259	Minor gas vesicle protein of unknown function
GvpJ	11,983	Minor gas vesicle protein similar to GvpA and GvpM (pfam741)
GvpK	12,695	Predicted gene product with slight similarity to GvpC
GvpL	31,994	Minor gas vesicle protein with coiled-coil domain, laddering (pfam 6386)
GvpM	9248	Minor gas vesicle protein similar to GvpA and GvpJ (pfam741)
GvpN	39,228	Minor gas vesicle protein with NTP/AAA+ family motif
GvpO	13,232	Minor gas vesicle protein with regulatory or structural function

**Figure 2:**
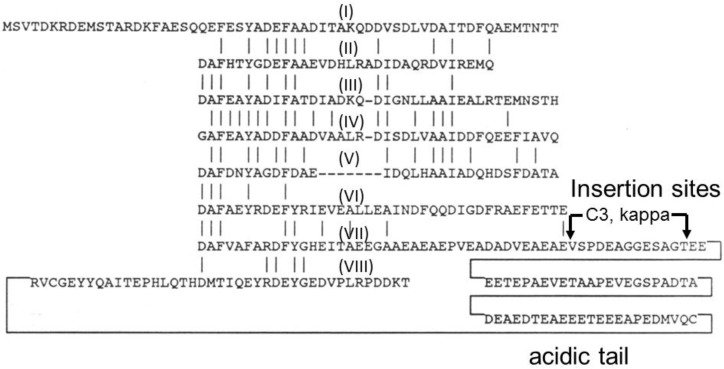
Amino acid sequence of GvpC from *Halobacterium* sp. NRC-1 showing internal repeats (numbered by Roman numerals I–VIII) with identities indicated by vertical lines. The location of insertion sites (C3 and *kappa*) used for gene fusions, and acidic tail near the C-terminal region are labeled.

The *Halobacterium* sp. NRC-1 GvpC protein was also readily detected in GVNP preparations by immunoblotting (Figure 2) [40]. GvpC protein was released under mildly denaturing conditions, weakening the nanoparticles and suggesting the involvement of the protein in strengthening GVNPs by binding to the external surface of the membrane in *Anabaena flos-aquae* [39,78]. Similar results have been obtained for GVNPs from *Halobacterium* sp. NRC-1 [79]. The GvpC protein family contains a motif that is repeated 4–5 times in cyanobacteria [45,80,81] and eight times in haloarchaea [40,41]. These repeats are hypothesized to bind to multiple copies of GvpA on the external surface of the GVNP membrane. An acidic tail is present in the *Halobacterium* GvpC protein, which is speculated to be important for stability under the highly saline conditions in which they are found [82,83]. Interestingly, GVNPs could be produced in *Halobacterium* sp. NRC-1 in which the acidic tail of GvpC was fused with antigenic peptides and proteins, resulting in the display of antigens on the nanoparticles [5,6,7,8,9,10,11,12].

Three additional proteins, GvpF, GvpG, and GvpL, were observed in *Halobacterium* NRC-1 GVNPs using antisera against unique peptides from these proteins (Table 1) [13]. GvpF and GvpL (pfam 6386) contain coiled-coil domains, suggestive of self-association and nucleation [84]. In addition, the GvpL protein showed laddering on gels [13]. Recently, the structure of GvpF from the cyanobacterium *Microcystis aeruginosa* was determined using X-ray crystallography and the protein was detected by immunogold staining on the internal surface in the cone regions of the nanoparticles, consistent with its function in initiation of GVNP synthesis [85].

The functions of the other *gvp* gene products remain unclear at present (Table 1). GvpD and GvpN sequences contain nucleotide binding motifs, consistent with a role in an energy requiring step, although the former has been proposed to be a regulatory protein and the latter to be structural [41,50,73,86]. GvpI, which was also detected in GVNPs by mass spectrometry, represents the only predicted basic protein, one of very few found in the highly acidic *Halobacterium* sp. NRC-1 proteome [13,41,48,73]. The roles of the other *gvp* gene products and proteins in GVNP biogenesis are not yet clear.

## 5. Antigen Display on Gas Vesicle Nanoparticles

*Halobacterium* sp. NRC-1 GVNPs have been described as self-adjuvanting, since they elicit immune responses without the addition of exogenous adjuvant [5]. Immunization with wild-type GVNP results in immunostimulation (Table 2), without any ill effects observed in mice, either in terms of the injection site or their survival [5,6]. These findings together with genetic studies showing that foreign sequences inserted into the *gvp*C gene in *Halobacterium* sp. NRC-1 could be tolerated, prompted the development of GVNPs for antigen display [87,88]. An initial key observation was that a plasmid, pFL2*gvp*C::κΔ, containing the entire *gvp* gene cluster with an 18 base pair insertion of foreign DNA near the C-terminus of the *gvpC* gene produced nanoparticles in *Halobacterium* [42]. Analysis of the resulting GVNPs showed the production of nanoparticles displaying the encoded six-amino acid peptide, ESSGTF, using ELISA assays (Table 2) [5]. When BALB/c mice were immunized IP with the recombinant nanoparticles, IgG and IgM levels were found to be elevated, and after re-immunization eight months post first boost, peptide-specific antibody titers measured increased rapidly, indicating that the recombinant GVNPs elicited long-term humoral responses (Table 2). These experiments were conducted without the addition of any exogenous adjuvant, indicating that the recombinant GVNPs served as both carrier and adjuvant.

**Table 2:** Antigen display on GVNPs and immune responses.

Antigen Displayed	*Halobacterium* sp. strain(s) designation	Antigen AA Length(s)	Administration Route(s)	Response(s)
None (Wild-type)	NRC-1 (pFM104d) or SD109 (pMS104)	N/A	IP, CC	TNF-α, IL-6, IL-12, TLR4, TLR5
TNP hapten	NRC-1	N/A	IP	IgG
Hexapeptide ESSGTF	SD109 (pFL2*gvp*C::κΔ)	6	IP	IgM, IgG
SIV Gag fragments	SD109 (pFM101d::51, 504, 705)	17, 168, 235	IP	IgG, mB-cell activation
SIV Tat, Rev, Nef1	SD109 (pMS104d::*tat, rev, nef1*)	50, 81, 214	SC	IgG1, mB-cell activation, IL-10, IL-12, IL-18 (except Nef1)
*C. trachomatis* MOMP fragments VD3, VD4	SD109 (pMS104::VD3 *, VD4)	48, 69	CC	TNF-α, IL1-β *, IL-6, IL-12, TLR4, TLR5 *
*C. trachomatis* OmcB fragments 23, 420	SD109 (pMS104::OmcB23 ^†^, B420)	162, 144	CC	TNF-α, IL-6, IL-12, TLR4, TLR5 ^†^
*C. trachomatis* PompD fragments M1, N2	SD109 (pMS104::PompDM1, DN2 ^‡^)	173, 222	CC	TNF-α, IL-6 ^‡^, IL-12 ^‡^, TLR4
*S. enterica* SopB fragments 4, 5	SD109 (pSD104::SopB4, B5)	101, 167	IP (B5 only)	CD4^+^ T-cells, IFNγ, IL-2, IL-9
*P. falciparum* CSP	SD109 (SD*csp*20), Δ*ura*3Δ*gvp*C (pDRK*csp*6)	398	N/T	N/T
*P. falciparum* Enolase	SD109 (SD104::Eno)	15	IP	IgG
*G. princeps* Luciferase	NRC-1, Δ*ura*3Δ*gvp*C (pDRKC3-*luc*)	185	N/T	N/T

NOTES: *Halobacterium* sp. marked with *, ^†^, or ^‡^ produce additional cytokines marked with the same symbols (*cf.* columns labeled “*Halobacterium* sp.” and “Responses”). AA, amino acids; CC, cell culture; IP, intraperitoneal; N/A, not applicable; N/T, not tested; SC, subcutaneous.

Following these promising results, GVNPs were chemically cross-linked to the hapten, 2,4,6-trinitrophenyl (TNP), and BALB/c mice, were inoculated IP [5]. This resulted in TNP-specific antibody responses following primary immunization, even in the absence of any exogenous adjuvant (Table 2). The titers increased after two boosts, and after an eight-month hiatus, a strong, specific, and long-lived immune response with immunologic memory was detected.

In a study that involved the expression of viral antigens on the GVNPs, the nanoparticles were engineered to display polypeptides of 17–235 amino acids length from the Gag protein of the simian immunodeficiency virus (SIV) fused to the GvpC protein using the pSD104 precursor, pFM104d [6]. BALB/c mice inoculated IP with these recombinant GVNPs showed antibody response in the absence of external adjuvant (Table 2) [6]. The recombinant GvpC-SIV proteins were recognized by antibodies produced in SIV-infected monkeys, indicating that the Gag protein adopted a conformation recognizable to the immune system when fused to GvpC and displayed on GVNPs. There was a strong and rapid increase in IgG antibody response 10 days after the second booster, reflecting the activation of memory B cells. Antigen specific titers continued to remain high and immune memory was able to be stimulated for the lifespan of the animals.

A series of expression plasmid derivatives of pFM104d were constructed that contained fragments encompassing the entire SIV genome [7,8,89]. Of these, three were used to express SIV-GvpC fusion proteins, Tat (50 amino acids), Rev (81 amino acids), and Nef1 (214 amino acids) (Table 2). These were displayed as GvpC-fusions on the outer surface of the GVNPs and resulted in similar immunogenic responses as observed for the GvpC-Gag fragments, primarily an up-regulation of IgG1, with activation of memory B cells. Immunostaining and subsequent cytological observations using J774A.1 murine macrophages exposed to Gag, Tat, Rev, and Nef1 displayed on GVNPs showed that humoral responses included a slow natural release of antigenic epitopes over time, with the initial breakdown of the SIV peptides, followed by a slower breakdown of the larger GvpC and other GVNP peptides [6,7,8]. Macrophages took up and processed GVNPs displaying SIV proteins with degradation occurring over days. Cytokine release was determined using ELSIA assays and 12 h post exposure, IL-10 levels were up in Tat and Rev-GVNP exposed cells, and IL-18 levels were up in Nef1-GVNP exposed cultures. After 24 h, IL-18 levels in Tat- and IL-10 levels in Nef1-GVNP exposed cultures peaked. At 48 h, IL-12 levels were elevated in Rev- and Nef1-GVNP-exposed cells.

In a study involving the expression of antigens from an obligate intracellular human pathogenic bacterium, *Chlamydia trachomatis*, outer membrane proteins were expressed as GvpC-fusions on the GVNP surface using the *Halobacterium* antigen display system [9]. Sequences of fragments coding for the major outer membrane protein (MOMP), outer membrane complex B (OmcB), and polymorphic outer membrane protein D (PompD) of *Chlamydia* were fused to *gvp*C and proteins were displayed on GVNPs (Table 2). The protein fragments were detected using *Chlamydia*-infected patient sera, indicating that they adopted native conformations on the nanoparticles and were antigenic. A similar degree of stability and degradation of the *Chlamydia* antigens displayed on GVNPs were observed, as for displayed SIV antigens. Immunostaining of human foreskin fibroblast cultures exposed to the *Chlamydia* antigen-GVNPs showed that the nanoparticles were taken up into the cells, and accumulated in a focused locus. The nanoparticles were gradually broken down and the antigenic fragments, starting with the *Chlamydia* antigens, were transported and displayed on the cell surface. The immunological responses were determined using RT-PCR and showed TLR-4 and -5 engagement and TNF-α, IL-1β, IL-6, and IL-12 production.

Using a bioinformatic approach, antigens were evaluated from an enteric bacterium, *Salmonella enterica*, a facultative intracellular pathogen [90,91]. SopB, a secreted inosine phosphate effector protein injected by bacteria during infection into the host cell, was selected for GVNP display [10,92]. Two SopB fragments, 100–165 amino acids in size, were produced from the fusion of synthetic gene fragments to *gvp*C (Table 2). The gene fragments were codon-optimized for optimum expression in *Halobacterium* sp. NRC-1 [10,93]. The proper conformation of the displayed antigens on GVNPs was shown using antigen-specific antisera. When SopB5-GVNPs were used for IP boosts following priming with an attenuated *S.* Typhimurium, a strong and long-lived immune response was observed. Serum cytokine responses showed increases in the pro-inflammatory cytokine, IFN-γ, IL-2, and IL-9, indicating that the Th1 pathway known to be involved in the response to intracellular *Salmonella* pathogens is induced.

In the first ever challenge experiment using GVNP-vaccine candidates, following immunizations and boosting, mice were challenged orally with 10^7^ virulent *Salmonella*, and the MLN, liver, and spleen were isolated one week post-challenge. In each of the three organs, bacterial loads were lower by at least two orders of magnitude for mice immunized with SopB5-GVNPs [10]. Bacterial load in the spleen was also significantly reduced in SopB4-GVNP immunized mice. CD4^+^ T-cells were also found to be elevated 2–4-fold in the spleen of mice immunized with SopB5-GVNPs compared to wild-type [10]. Further challenge experiments were conducted to determine protection, and mice immunized with SopB5-GVNPs survived several days longer than those immunized with wild-type GVNPs [11]. These encouraging results with SopB-GVNPs were the first and only, to date, to illustrate the potential efficacy of GVNPs in disease prevention and survival after real-life challenge.

Recently, *Plasmodium falciparum* antigens, including circumsporozoite protein (CSP), the major outer coat protein in sporozoites, and enolase, a glycolytic enzyme with moonlighting functions expressed both intracellularly and extracellularly in several stages of the parasite lifecycle, have been displayed on GVNPs [12,94]. The *Halobacterium* expression system was reported to be capable of expressing the entire CSP molecule, freely as well as a fusion to the GvpC protein, suggesting that it can be successfully displayed on the nanoparticles. In a different approach, a small, highly conserved parasite-specific epitope of enolase was displayed on GVNPs and found to exhibit protective properties against the rodent form of the disease. These findings may provide significant advantages in the development of an effective vaccine against malaria, one of the most common infectious diseases worldwide.

## 6. GVNP Bioengineering System

An improvement to the genetic system for bioengineering for facilitating display of antigens on GVNPs has recently been reported [92]. The original system employed large plasmids (pFL2, pFM104d, and pSD104) containing the entire GVNP gene cluster and utilized a host strain deleted for all of the *gvp* genes (SD109) [5,6,15]. In the recently developed bioengineering system, a series of smaller, more easily engineered plasmid expression vectors were constructed, containing a fragment of *gvp*C expressed from the high-level, cold-inducible, *gvp*A promoter [92,95,96]. Foreign sequences were easily incorporated to produce GvpC fusions for display on the GVNP surface. A *gvp*C deletion strain, *Halobacterium* Δ*ura*3Δ*gvp*C, was also constructed using the *ura*3-based gene deletion method to serve as the expression host [92,95].

**Figure 3:**
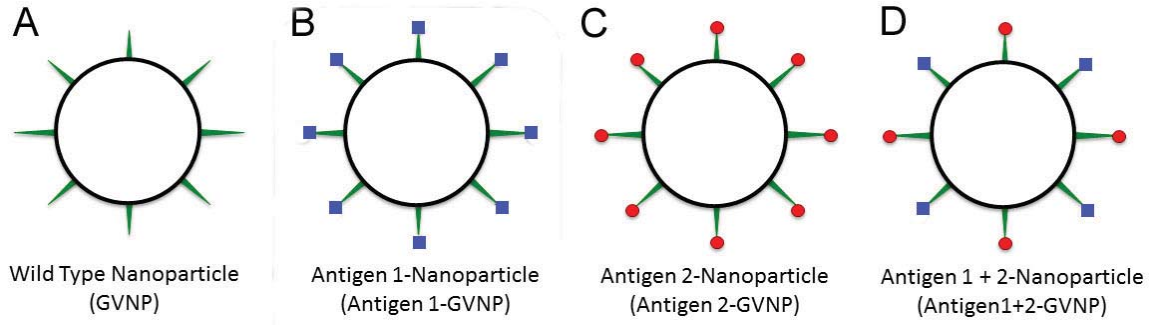
Antigen display employing GVNPs. Wild-type GVNPs (black circle, (**A**)) can be engineered to display, via fusions to GvpC (green spikes), different antigens (blue squares or red balls) separately (**B**,**C**) or together (**D**) on nanoparticles.

The new genetic system was tested by inserting a synthetic gene encoding the reporter protein, luciferase, from the marine arthropod *Gaussia princeps* into the expression vector (Table 2). The luciferase protein was expressed as a fusion protein with GvpC and was displayed in an enzymatically active form on GVNPs [92]. The strain containing both the wild-type and GvpC fusion forms of the *gvp*C gene, produced GVNPs displaying both the GvpC and GvpC-luciferase fusion proteins, indicating that multiple GvpC protein types may be displayed simultaneously on the surface of GVNPs (Figure 3). The capability to express multiple GvpC proteins together on individual GVNPs, including active enzymes like luciferase, is likely to be useful for a variety of diverse biotechnological applications, such as for generating a “cocktail” mix of antigens for potentially improved multivalent protective vaccines.

## 7. Future Opportunities and Challenges

Future prospects for use of GVNPs as an antigen display system and for vaccine development seem promising, although significant challenges still remain. Clearly, continued advancements in the methodology for display of antigenic peptides and proteins on GVNPs will facilitate the development and testing of potential vaccine candidates and may allow their use in other biotechnological applications. The availability of a streamlined vector for *gvp*C fusions and a host system for expression of diverse antigens is key to future efforts [92].

Although available studies on GVNPs have shown that they are easily bioengineered, self-adjuvanting and highly immunogenic, as well as biocompatible, shelf-stable, and non-toxic, a number of important fundamental studies remain to be carried out in future. For example, their extraordinary stability has precluded the determination of the precise structure, composition, and biogenesis of the nanoparticles [13,14,15,16]. As a result, the roles of many GVNP proteins are not yet clear. Important questions remain regarding the steps in initiation of organelle biosynthesis and the transition of the structures from cones to cylinders. The proteins involved in scaffolding, chaperoning, turnover, and degradation, if they exist, are unknown. In addition to these fundamental questions, the precise density and conformation of GvpA, GvpC, and other proteins, including displayed antigens, have not been determined. As a result, considerable fundamental work still needs to be conducted to take full advantage of biomedical and biotechnological opportunities.

While the self-adjuvanting properties of GVNPs are known, detailed studies of the immunogenicity of GVNPs have not yet been fully conducted [5,6,7,8,9,10,11,12]. Moreover, the use of exogenous adjuvants for further increasing immunogenicity of the isolated nanoparticles has not been explored. Different modes of delivery and the degree of stability of GVNPs in animals have also not been carefully compared. A particularly important question is whether antigens may be delivered to the gut mucosa via oral immunization. Given the non-toxicity of the host cells and their propensity for hypotonic lysis, the potential for oral delivery of GVNPs in cells desiccated with salt is of significant interest. Moreover, demonstration that a GVNP-based vaccine may offer full protection from challenge with pathogens remains a high priority [11].

The ability to display multiple proteins, including active enzymes and other potentially therapeutic proteins, may allow the development not only of more effective vaccines, but also other innovative biomedical and biotechnological applications. Targeting specific cell types, e.g., cancer cells, and delivering therapeutic agents to specific regions of the body, are valuable future goals. To realize the full potential of these nanoparticles, it is especially important to understand the biogenesis of GVNPs in order to bioengineer additional useful capabilities, such as encapsulation of therapeutic agents, and determining the size and shape of the nanoparticles. While significant challenges remain for clinical utilization of GVNP technology, continued expansion of our understanding of these unique natural protein nanoparticles is likely to lead to the development of new uses and applications.

## 8. Conclusions

Studies of GVNP genes and proteins in the salt-loving microorganism, *Halobacterium* sp. NRC-1, are facilitating the biotechnological application of the novel nanoparticles to antigen delivery and vaccine development. Protective antigens from viral, bacterial, and eukaryotic pathogens fused to the GvpC protein and displayed on GVNPs have shown promising results. The buoyancy and stability of GVNPs are novel properties valuable for a variety of processes, including facile purification by flotation and distribution without cold storage. More detailed understanding of GVNPs and their biogenesis are likely to further expand opportunities for bioengineering these nanoparticles and their development as a platform for vaccines and drug delivery. Translational biomedical applications are likely to increase the value of biocompatible gas vesicle nanoparticles as a benefit to society.
